# The Predictive Value of Pre-operative N-Terminal Pro-B-Type Natriuretic Peptide in the Risk of Acute Kidney Injury After Non-cardiac Surgery

**DOI:** 10.3389/fmed.2022.898513

**Published:** 2022-06-16

**Authors:** Xiang-Bin Liu, Ke Pang, Yong-Zhong Tang, Yuan Le

**Affiliations:** Department of Anesthesiology, Third Xiangya Hospital, Central South University, Changsha, China

**Keywords:** pre-operative N-terminal pro-B-type natriuretic peptide, post-operative acute kidney injury, prediction model, non-cardiac surgery, cohort study

## Abstract

**Objective:**

To evaluate the association between N-terminal pro-B-type natriuretic peptide (NT-proBNP) and risk of post-operative acute kidney injury (PO-AKI).

**Methods:**

The electronic medical records and laboratory results were obtained from 3,949 adult patients (≥18 years) undergoing non-cardiac surgery performed between 1 October 2012 to 1 October 2019 at the Third Xiangya Hospital, Central South University, China. Collected data were analyzed retrospectively.

**Results:**

In all, 5.3% (209 of 3,949) of patients developed PO-AKI. Pre-operative NT-proBNP was an independent predictor of PO-AKI. After adjustment for significant variables, OR for AKI of highest and lowest NT-proBNP quintiles was 1.96 (95% CI, 1.04–3.68, *P* = 0.008), OR per 1-unit increment in natural log transformed NT-proBNP was 1.20 (95% CI, 1.09–1.32, *P* < 0.001). Compared with clinical variables alone, the addition of NT-proBNP modestly improved the discrimination [change in area under the curve(AUC) from 0.82 to 0.83, ΔAUC=0.01, *P* = 0.024] and the reclassification (continuous net reclassification improvement 0.15, 95% CI, 0.01–0.29, *P* = 0.034, improved integrated discrimination 0.01, 95% CI, 0.002–0.02, *P* = 0.017) of AKI and non-AKI cases.

**Conclusions:**

Results from our retrospective cohort study showed that the addition of pre-operative NT-proBNP concentrations could better predict post-operative AKI in a cohort of non-cardiac surgery patients and achieve higher net benefit in decision curve analysis.

## Introduction

Acute kidney injury (AKI) is a common complication in hospitalized patients and very common in critically ill patients (1), which is associated with adverse outcomes including death (2–4). The pooled incidence of AKI after cardiac surgery is 22.3% (5), whereas that after major abdominal surgery is 13.4% (6). Post-operative AKI (PO-AKI) might also increase the length of hospital stay and healthcare costs (7), so there has been heightened interest in early recognition of PO-AKI. Promoting the early prediction of PO-AKI would help early recognition of high-risk patients, facilitate the pre-operative and perioperative management to prevent iatrogenically induced AKI events, and ameliorate surgical outcomes.

Several pre-operative risk prediction models for PO-AKI following noncardiac surgery have been developed and provided moderate levels of accuracy in both adults and children (8–13). Recent studies have associated cardiac biomarkers B-type natriuretic peptide (BNP) and N-terminal pro-BNP (NT-proBNP) with risk of AKI in several medical settings (14–18) as well as after surgery (19–21). BNP is a cardiac hormone secreted mainly by the cardiac ventricles in response to myocardial stretch as a result of volume overload and its biological effects include diuresis, natriuresis, and arterial and venous dilatation. BNP is currently being used to guide medical therapy for heart failure and has been added to several algorithms for perioperative risk stratification for mortality and cardiopulmonary complications (22). Early in 2008, Elíasdóttir et al. (23) had found that serum NT-proBNP level was higher in patients who developed acute renal failure after cardiac surgery. Further studies demonstrated the predictive value of serum NT-proBNP in PO-AKI after cardiac surgery both in adults and children (19, 20, 24). But, the investigations on the predictive value of serum NT-proBNP in PO-AKI after non-cardiac surgery are rare (21, 25–27). Patients undergoing cardiac surgery usually experience significant haemodynamic instability and severe PO-AKI is common (19, 26). But patients receiving noncardiac surgery are generally in stable conditions, have lower BNP/NT-proBNP level and most PO-AKIs are mild (26). The mechanisms of renal injury might be different. Therefore, the predictive ability of BNP/NT-proBNP for AKI in the noncardiac surgical cohort remains unclear. This study aims to test the value of pre-operative NT-proBNP in PO-AKI risk identification in non-cardiac surgery settings.

## Methods

### Design and Selection Criteria

This retrospective study was performed at the Third Xiangya Hospital, Central South University. Ethics approval for this study was provided by the ethics committee of Third Xiangya Hospital, Central South University (F-22002). Because it is a retrospective observatory study, written informed consent was not applicable. The protocol was registered in the Chinese Clinical Trial Registry (ChiCTR2200055942).

The study cohort was identified using the hospital's Electrical Medical Record System. Adult (age ≥18 years) patients who had a serum creatinine (Scr) and NT-proBNP measurement within 30 pre-operative days and at least one Scr measurement within 7 days after surgery from 1 October 2012 to 1 October 2019 were included. The exclusion criteria were organ transplant surgery, cardiac and urological surgery, pre-operative acute myocardial infarction, dialysis, systolic blood pressure before anesthesia <90 mmHg and estimation glomerular filtration rate (eGFR) <15 ml min^−1^ 1.73 m^−2^.

### Data Collection

The following information was collected: (1) Baseline information including age, sex and body mass index (BMI); (2) individual history including pre-operative complications and medical history (hypertension, diabetes mellitus, coronary artery disease, stroke, renal disease, ascites, hyperlipidemia, use of renin-angiotensin-aldosterone system inhibitors); (3) laboratory data including pre-operative serum hemoglobin, direct bilirubin, creatinine and eGFR calculated using the CKD Epidemiology Collaboration formula (28); (4) intraoperative data including emergency surgery, surgical grade, type and duration of surgery, blood pressure before anesthesia, anesthesia method, ASA grade, amount of fluid infusion and out and American Society of Anesthesiologists' (ASA) physical status and (5) post-operative outcomes such as the occurrence of AKI. All measurements were carried out in accordance with the Declaration of Helsinki.

Serum NT-proBNP concentrations were measured by ReLIA® multi-function Immunodetector (Shenzhen, PRC) in the Laboratory of Cardiology in the Third Xiangya Hospital. For patients who had multiple pre-operative NT-proBNP measurements, the lowest value was used.

The primary outcome was post-operative AKI, defined according to the Kidney Disease: Improving Global Outcomes (KDIGO) 2012 creatinine criteria (29), as either of the following: an increase in Scr by ≥0.3 mg dl^−1^ within 48 h or a≥1.5 times increase in Scr from baseline within 7 post-operative days. The baseline Scr level was the lowest measure within pre-operative day 7.

### Statistical Analysis

The sample size had not been calculated in this study because all eligible patients were analyzed to maximize statistical power. All statistical analyses were performed by R4.1.0 (http://cran.r-project.org).

Continuous variables were presented as mean (Standard Deviation, SD)and categorical variables as counts and percentage. Mann–Whitney *U*-test was used to compare continuous variables between groups, whereas the χ^2^ test or Fisher's exact test was used for categorical variables as appropriate.

Because pre-operative NT-proBNP concentrations were skewly distributed, natural logarithmic (ln) transformation was performed. Univariable and multivariable logistic regression analysis were conducted to clarify the association between pre-operative NT-proBNP and PO-AKI. NT-proBNP was treated as categorical (in quintiles) and continuous variables (ln transformed). In the multivariable model, significant baseline variables mentioned before were used as adjusted factors for post-operative AKI. Results were presented as odds ratio (OR) and 95% confidence interval (CI).

The restricted cubic splines to the continuous model were used to further analyse the multivariable association between pre-operative NT-proBNP and PO-AKI.

In order to compare the model's ability to discriminate AKI cases from non-AKI cases, DeLong's method was used to assess the change in area under the receiver operating characteristic curves (AUCs) (30).

The risk reclassification ability was valued by the net reclassification improvement (NRI) and the integrated discrimination improvement (IDI) indices. Furthermore, the possibility that whether pre-operative NT-proBNP could improve the predictive ability of two AKI pre-operative prediction models in noncardiac surgical patients, i.e., the weighted general surgery AKI (GS-AKI) risk index and the simple post-operative AKI risk (SPARK) index was tested. Finally, the decision curve analysis (DCA) was applied to assess the practical usefulness of different models.

## Results

**Figure 1:**
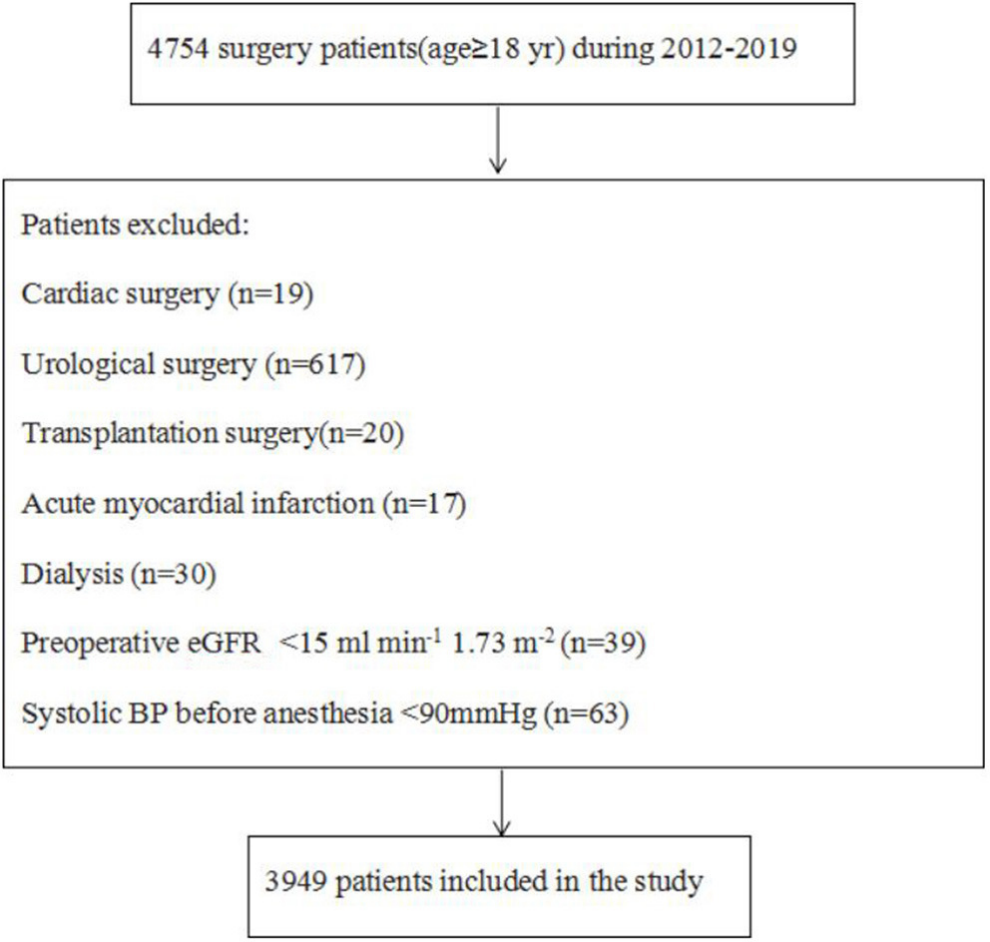
Flow chart for patient selection.

The patient selection process was shown in Figure 1. 209 out of the 3,949 patients (5.3%) eligible for analysis developed AKI within 1 week after surgery. Characteristics of the study cohort were shown in Table 1 and Supplementary Table S1. Those older, with stroke or renal diseases were more likely to be complicated with post-operative AKI.

**Table 1:** Baseline characteristics of the study.

**Variables^a^**	**Overall**	**No AKI**	**AKI**	***P***
*n*	3949	3740	209	
Age, years	62.57 (13.48)	62.39 (13.35)	65.74 (15.29)	<0.001
Male (%)	2428 (61.5)	2337 (62.5)	91 (43.5)	<0.001
BMI kg m^−2^	23.77 (7.13)	23.83 (7.26)	22.68 (4.26)	0.023
**Medical history and meditations 1 week before surgery**
Hypertension (%)	1922 (48.7)	1806 (48.3)	116 (55.5)	0.05
Stroke (%)	468 (11.9)	416 (11.1)	52 (24.9)	<0.001
ACEI (%)	116 (2.9)	103 (2.8)	13 (6.2)	0.007
Diuretics (%)	175 (4.4)	144 (3.9)	31 (14.8)	<0.001
Beta blocker (%)	120 (3.0)	107 (2.9)	13 (6.2)	0.011
Anti-platelet and anticoagulant (%)	401 (10.2)	355 (9.5)	46 (22.0)	<0.001
NSAIDs (%)	629 (15.9)	617 (16.5)	12 (5.7)	<0.001
**Pre-operative findings**
Ln NT-proBNP^b^	5.38 (1.92)	5.31 (1.91)	6.63 (1.68)	<0.001
NT-proBNP^c^, ng L^−1^	266 [118 to 698]	255 [114 to 641]	882 [307 to 2158]	<0.001
Creatinine, mg dl^−1^	0.81 (0.36)	0.80 (0.34)	0.97 (0.58)	<0.001
Hb, g L^−1^	119.57 (21.90)	120.22 (21.53)	107.83 (25.01)	<0.001
ALB, g L^−1^	38.60 (5.46)	38.82 (5.32)	34.54 (6.24)	<0.001
ASA (%)				<0.001
2	1447 (36.6)	1429 (38.2)	18 (8.6)	
3	2003 (50.7)	1883 (50.3)	120 (57.4)	
4	427 (10.8)	362 (9.7)	65 (31.1)	
5	18 (0.5)	12 (0.3)	6 (2.9)	
**Surgical characteristics**
Trans-abdominal (%)	2201 (55.7)	2090 (55.9)	111 (53.1)	0.475
Emergency surgery (%)	525 (13.3)	452 (12.1)	73 (34.9)	<0.001
Duration, min.	157.68 (88.55)	157.03 (88.43)	169.36 (89.98)	0.05
Fluid loss, ml	656.80 (530.32)	658.24 (528.97)	630.91 (554.60)	0.468
Blood loss, ml	257.66 (431.54)	252.17 (428.01)	355.86 (481.09)	0.001

*Values are mean (SD) or number (%)*.

a *Categorical variables were shown as counts (percentages) and continuous variables as means (standard deviation)*.

b *In transformed serum NT-proBNP*.

c *Serum NT-proBNP concentration [median, interquartile ranges]*.

*AKI, acute kidney injury; BMI, Body Mass Index; ACEI, angiotensin converting enzyme inhibitors; NSAIDs, non-steroidal anti-inflammatory drugs; Hb, hemoglobin; ALB, albumin; ASA, American Society of Anesthesiologists; NT-proBNP, N-terminal pro-B-type natriuretic peptide*.

The median (interquartile range, IQR) pre-operative NT-proBNP was 266.28 (117.92–698.18) ng L^−1^. pre-operative ln NT-proBNP concentrations were markedly higher in patients who developed AKI after surgery than in those who did not (median 255.08 vs. 882.44 ng L^−1^, *P* < 0.001, Table 1). Among various surgery types, neurosurgery had highest NT-proBNP concentration and also highest incidence of PO-AKI, whereas, gynecological surgery had the lowest ones (median 898.14 vs. 158.23 ng L^−1^, 10.36 vs. 1.39%, Table 2). The *P* for trend test demonstrated that the risk of AKI increased along with the pre-operative NT-proBNP quintile (*P* for trend < 0.00001). The odds of AKI for the highest quintile were eight times higher than that with the lowest (Table 3). After adjustment by patient demographics as age and gender, medical history of hypertension and pre-operative use of NSAIDs, ACEI, diuretics, receptor blockers and anti-platelets and coagulant, laboratory findings as serum creatinine, hemoglobin and albumin and surgical characteristics as ASA physical status, surgery duration, blood loss and fluid loss in a logistic regression model, quintiles 4 and 5 still correlated independently with post-operative AKI (*P* < 0.05). When included in the logistic model, pre-operative NT-proBNP was also significantly and independently associated with PO-AKI risk [adjusted OR per 1-unit increment in ln (NT-proBNP) 1.20, 95% CI, 1.09–1.32, *P* < 0.001, Table 3].

**Table 2:** The relationship between pre-operative NT-proBNP measurement and development of AKI in different types of surgery.

**Type of surgery**	**Overall *N* (%)**	**AKI *N* (%)**	**Incidence of AKI (%)**	**NT-proBNP ng L^−1^ median (IQR)**	**Univariate analysis OR (95% CI)**	**Multivariate analysis OR (95% CI)**
General surgery	1624 (41.1)	87 (41.6)	5.36	271.81 (127.84, 642.43)	1.84 (1.56–2.19)	1.39 (1.16–1.68)
Gynecology	790 (20.0)	11 (5.3)	1.39	158.23 (73.69, 347.79)	1.54 (0.89–2.25)	1.75 (0.74–3.44)
Orthopedics	728 (18.4)	45 (21.5)	6.18	300.75 (124.47, 873.79)	1.36 (1.16–1.59)	1.27 (1.01–1.59)
Neurosurgery	308 (7.8)	31 (14.8)	10.06	698.14 (269.00, 1548.60)	1.32 (1.09–1.62)	1.14 (0.86–1.48)
Other	499 (12.6)	35 (16.7)	7.01	309.19 (138.19, 993.35)	1.42 (1.22–1.68)	1.27 (1.01–1.59)

*AKI, acute kidney injury; NT-proBNP, N-terminal pro-B-type natriuretic peptide; IQR, interquartile range; OR, odds ratio; 95% CI, 95% confidence interval*.

**Table 3:** The *P* trend test for the correlation of pre-operative N-terminal pro-B-type natriuretic peptide with acute kidney injury after noncardiac surgery.

	**Categorized NT-proBNP (quintiles)**						**Continuous ln (NT-proBNP)^b^**
**OR**	** <92**	**92–190**	**190–387**	**387–894**	**>894**	***P*** **-trend**	
Unadjusted	1.0	1.81 (0.94, 3.51)	1.74 (0.90, 3.39)	3.27 (1.78, 6.02)	8.22 (4.66, 14.51)	<0.001	1.54 (1.42, 1.67)
Adjusted^a^	1.0	1.57 (0.79, 3.12)	1.04 (0.52, 2.08)	1.36 (0.71, 2.61)	1.96 (1.04, 3.68)	0.007	1.20 (1.09, 1.32)

*Values are OR (95% CI)*.

a *Adjusted for age (per year), sex, ASA physical status, hypertension, Scr, use of NSAIDs, ACEI, diuretics, beta blockers and anti-platelets and coagulant, surgery duration, blood loss, fluid loss, serum hemoglobin and serum albumin*.

b *Per 1-unit increment*.

*ASA, American Society of Anesthesiologists; ln, natural log-transformed; NT-proBNP, N-terminal pro-B-type natriuretic peptide*.

**Figure 2:**
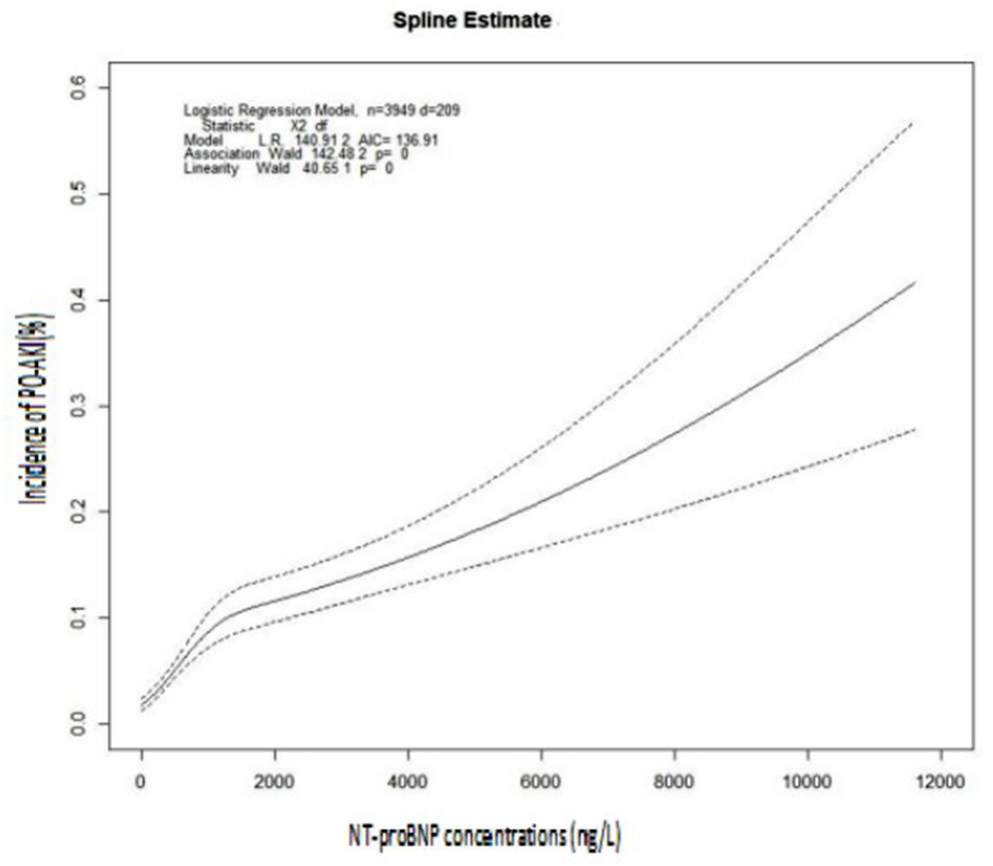
Restricted cubic spline regression of the association between NT-proBNP and post-operative AKI. The incidence of PO-AKI (*y*-axis) as a function of pre-operative NT-proBNP concentrations (ng L^−1^, *x*-axis). The dotted line indicates 95% CIs. There was a positive correlation between pre-operative NT-proBNP concentrations and the incidence of PO-AKI.

The multi-variable adjusted OR from the restricted cubic spline models for post-operative AKI by pre-operative NT-proBNP was showed in Figure 2. The discrimination measure AUC was 0.71 (95% CI, 0.68–0.75) for the prediction model using only NT-proBNP, 0.82 (95% CI, 0.79–0.84) for one of multiple variable only model and 0.83 (95% CI, 0.80–0.85) for that containing both multiple variable and NT-proBNP. The addition of NT-proBNP to the model of multiple variable model slightly increased the predictive efficiency (Table 4). Furthermore, by adding of pre-operative NT-proBNP to the GS-AKI risk index or the SPARK index, the significant increase of AUC (*P* < 0.001), the continuous NRI and the IDI indices demonstrated the moderately upgrade of AKI prediction (Table 4).

**Table 4:** Comparison of acute kidney injury prediction models with and without pre-operative N-terminal pro-B-type natriuretic peptide.

	**Base model**	**Base model + NT-proBNP^a^**
**Multivariables logistical regression mode as base model** ^b^
AUC	0.82 (95% CI, 0.79 to 0.84)	0.83 (95% CI, 0.80 to 0.85)
ΔAUC	Reference	0.01, *P* = 0.024
NRI for event	Reference	−0.13 (95% CI, −0.26 to 0.005)
NRI for nonevent	Reference	0.28 (95% CI, 0.25 to 0.31)
NRI	Reference	0.15 (95% CI, 0.01 to 0.29)
IDI	Reference	0.01 (95% CI, 0.002 to 0.02)
**Weighted general surgery AKI risk index GS-AKI as the base model**
AUC	0.66 (95% CI, 0.62 to 0.70)	0.74 (95% CI, 0.71 to 0.78)
ΔAUC	Reference	0.08, *P* <0.001
NRI for event	Reference	−0.15 (95% CI,−0.28 to−0.01)
NRI for nonevent	Reference	0.64 (95% CI, 0.62 to 0.67)
NRI	Reference	0.49 (95% CI, 0.36 to 0.63)
IDI	Reference	0.04 (95% CI, 0.02 to 0.05)
**Simple Post-operative AKI risk index (SPARK) as the base model**
AUC	0.69 (95% CI, 0.65 to 0.72)	0.76 (95% CI, 0.73 to 0.80)
ΔAUC	Reference	0.07, *P* <0.001
NRI for event	Reference	−0.19 (95% CI, −0.32 to −0.05)
NRI for nonevent	Reference	0.64 (95% CI, 0.62 to 0.67)
NRI	Reference	0.46 (95% CI, 0.32 to 0.60)
IDI	Reference	0.03 (95% CI, 0.02 to 0.05)

a *Natural log-transformed NT-proBNP*.

b *Adjusted for age (per year), gender, hypertension, use of ACEI and anti-platelet and anticoagulant, nonelective surgery, surgery type, surgery duration, anesthesia type, hemoglobin and serum albumin*.

*AUC, change in area under the curve; AKI, acute kidney injury; ASA, American Society of Anesthesiologists; AUC, area under the curve; IDI, integral discrimination improvement; NRI, net reclassification improvement; NT-proBNP, N-terminal pro-B-type natriuretic peptide*.

**Figure 3:**
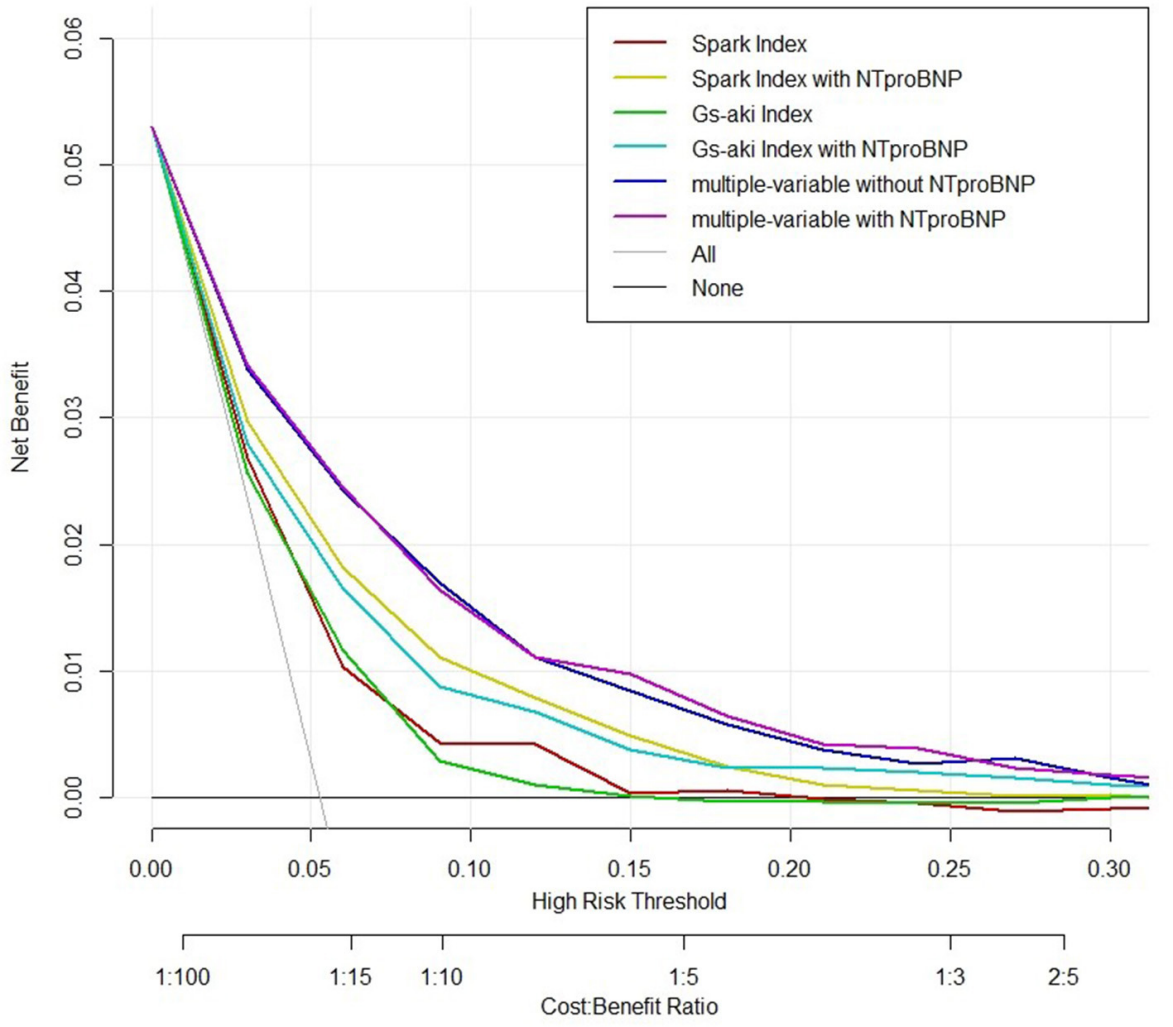
Decision curve analysis. Decision curve was developed for evaluating the net benefit of using models with or without pre-operative NT-proBNP for preventive management decisions for PO-AKI. NT-proBNP, N-terminal pro-B-type natriuretic peptide; GS-AKI index, the weighted general surgery acute kidney injury risk index; SPARK index, Simple Post-operative AKI Risk index.

Decision curve analysis (DCA) demonstrated that the model of clinical risk factors plus NT-proBNP acquired the highest net benefit for decision thresholds from 10 ~27% (Figure 3). The thresholds were suitable in clinical applications and above the threshold, a patient would be considered as “vulnerable” for AKI. For thresholds below 10% (i.e., one patient with PO-AKI for every 10 patients receiving surgery), the net benefits were comparable for the multiple variable model, but were higher than the SPARK and GS-AKI indexes either with or without NT-proBNP. Beyond a clinical decision threshold of 27% (i.e., one patient with AKI for every 3.7 patients receiving surgery), addition of NT-proBNP brought little benefit.

## Discussion

In this study, we found that AKI occurred in 5.3% of patients undergoing non-cardiac surgery. The pre-operative NT-proBNP was an independent predictor of PO-AKI. The addition of NT-proBNP to GS-AKI and SPARK indices modestly improved the discrimination and reclassification of the AKI prediction model. For decision makers, the threshold probability at which the greatest net benefit achieving from the prediction model with NT-proBNP was between 10 and 27%.

In 2021, a joint consensus report of the Acute Disease Quality Initiative and PeriOperative Quality Initiative (2) defined PO-AKI as AKI occurring within 7 days of an operative intervention using the Kidney Disease Improving Global Outcomes (KDIGO) definition (29) of AKI. PO-AKI is a common complication of major surgery and usually strongly associated with short-term surgical complications and long-term adverse outcomes, including increased risk of chronic kidney disease, cardiovascular events and death (2). To date, no effective therapeutic intervention has been shown satisfactory in the management of PO-AKI, prevention of PO-AKI is critical for a better surgical outcome. In specific clinical settings, 20%−30% of AKI cases are considered to be preventable (31). For any prevention strategies to be effective, patients with high risk need to be identified before kidney insults result in kidney damage, and AKI needs to be diagnosed as early as possible (3).

BNP and NT-proBNP are well-known predictive factors for major cardiac events, however, recent researches have demonstrated the clinical and prognostic implications of BNP and NT-proBNP in predicting AKI in several medical settings, such as critical illness (18), severe burn (16), coronary heart disease (14) and cardiac surgery (19, 20), but, its relationship with AKI after noncardiac surgery had rarely been investigated. Till now, to our knowledge, only four studies have addressed this issue (21, 25–27). The study by Cardinale et al. included 2,179 patients solely undergoing lung cancer surgery, a 10% incidence of AKI was reported, and, among the independent predictors of AKI, serum Scr [area under the curve (AUC) 0.70 (95% CI 0.67–0.74)] and NT-proBNP [AUC 0.71 (95% CI 0.67–0.74)] provided the highest predictive accuracy, and their combination further significantly improved AKI prediction [AUC 0.74 (95% CI 0.71–0.77)] (25). Chae's study was mainly focused on patients after living donor liver transplantation (LDLT). Among 272 adult patients (≧19 years old) included, 22.4% suffered from AKI immediately after LDLT. Serum BNP level was useful for predicting the early development of AKI after LDLT (AUC 0.69) (21). Acarbas in his study mentioned the NT-proBNP level in patients undergoing sine surgery could be a valuable prognostic marker for several post-operative complications including acute renal failure (27). The study by Zhao included 7,248 patients undergoing non-cardiac surgery and the incidence of AKI was 6.1%. The addition of NT-proBNP to the model containing only conventional clinical variables significantly increased the discrimination (AUC 0.77) and reclassification (continuous NRI 0.21) of AKI and non-AKI cases, and achieved higher net benefit in decision curve analysis (26). Our results (AUC 0.83 and continuous NRI 0.15) was consistent with Zhao's findings and further consolidated the predictive value of NT-proBNP for PO-AKI in non-cardiac surgery settings. Our prediction model was better than GS-AKI and SPARKS. The reason might be the inclusion of intra-operative variables which had been proved to have effects on PO-AKI. The modestly improvement of the prediction efficiency suggested cautions on making general interpretations of the value of adding NT-proBNP to the prediction model of PO-AKI. The caution was further intensified by the logistical regression results of different surgery types.

Recent researches had revealed some new pre-operative urinary bio- markers to improve the prediction of AKI risk. Dickkopf-3 concentration could predict the development of PO-AKI in patients receiving cardiac surgery with an AUC of 0.78 (32). Urine tissue inhibitor of metalloproteinases 2 (TIMP-2) combined with insulin-like growth factor binding protein 7 (IGFBP7) sufficiently predict the risk of AKI in patients undergoing major abdominal surgery with an AUC of 0.85 (33). These biomarkers representing different pathophysiological pathways of surgery-associated AKI (26) and the combination of multiple markers showed greater model improvement and might evaluate different pathways for targeted intervention. Furthermore, a randomized clinical trial in patients after major abdominal surgery with an increased AKI risk demonstrated that early prediction of PO-AKI by urine TIMP-2 × IGFBP7 followed by implementation of KDIGO care bundle would reduce PO-AKI severity, post-operative creatinine increase, length of ICU and hospital stay in patients after major non-cardiac surgery (34).

Our data might provide some possible clinical benefits for non-cardiac surgical patients. Those patients with abnormal pre-operative NT-proBNP might be identified as high-risk, pre-operative and intraoperative prophylactic strategies recommended by the joint consensus of Acute Disease Quality Initiative and Perioperative Quality Initiative for PO-AKI (2) could be implemented as early as possible, such as discontinuing angiotensin-converting enzyme (ACE) inhibitors and angiotensin receptor blockers (ARBs) for a minimum of 24 h before surgery, dependent on the specific medication, the use of goal-directed hemodynamic therapy in high-risk patients to optimize volume status, blood pressure and cardiac output, maintaining an intraoperative mean arterial blood pressure (MAP) >65 mmHg. Moreover, the high-risk patients may benefit from a closer monitoring in the post-operative period, in order to early detect and treat AKI-associated adverse clinical conditions (25).

Several potential limitations of this study must be addressed. Firstly, our study used a retrospective cohort from a single hospital, the overall generalisability of our results remains uncertain. Secondly, the study cohort included might be biased. Those patients needed to have both NT-proBNP and pre- and post-operative Scr tests might have higher baseline cardiovascular and renal risks burden than the overall noncardiac surgical population. Thirdly, PO-AKI is a multi-factorial complication influenced by multiple contributing factors, including surgery itself. Diversity of patients in this retrospective cohort study might also affect the overall applicability of our results. Last but not least, we only used the Scr levels for diagnosing PO-AKI, risking the fact that some patients might have developed PO-AKI with only oliguria are not included in our study. Future well-designed multi-center prospective studies should validate the predictive value of pre-operative NT-proBNP and whether early detection of high-risk patients followed by prophylactic strategies will prevent PO-AKI and improve overall post-operative outcomes.

## Conclusions

Our retrospective cohort study of 3,949 noncardiac surgery patients showed that pre-operative NT-proBNP concentrations were associated independently with the risk of PO-AKI. The addition of pre-operative NT-proBNP concentrations could better predict PO-AKI than the logistic regression model of conventional clinical risk factors and currently available GS-AKI and SPARK prediction indices. Further studies are required to test whether the management based on NT-proBNP prediction model in clinics could reduce the incidence of PO-AKI.

## Data Availability Statement

The original contributions presented in the study are included in the article/Supplementary Material, further inquiries can be directed to the corresponding authors.

## Ethics Statement

This study was reviewed and approved by the Ethics Committee of the Third Xiangya Hospital, Central South University (F-22002). Because it was a retrospective observatory study, written informed consent was not applicable.

## Author Contributions

YL and YZT: design and leading of the study and revision of the manuscript. XBL: interpretation of data, preparation, and revision of the manuscript. KP and XBL: acquisition of subjects and/or data and analysis. All authors critically revised the manuscript for important intellectual content, read, and approved the final manuscript.

## Funding

This work was supported by the Xiangya Bigdata foundation, the Health Commission of Hunan Province Project (20201802 to YZT), the National Natural Science Foundation of China (81771169 to YL), and Hunan Provincial Science and Technology Department (2021JJ31008 to YL).

## Conflict of Interest

The authors declare that the research was conducted in the absence of any commercial or financial relationships that could be construed as a potential conflict of interest.

## Publisher's Note

All claims expressed in this article are solely those of the authors and do not necessarily represent those of their affiliated organizations, or those of the publisher, the editors and the reviewers. Any product that may be evaluated in this article, or claim that may be made by its manufacturer, is not guaranteed or endorsed by the publisher.
