# Corrigendum: The predictive value of pre-operative N-terminal pro-B-type natriuretic peptide in the risk of acute kidney injury after non-cardiac surgery

**DOI:** 10.3389/fmed.2022.1010604

**Published:** 2022-09-15

**Authors:** Xiang-Bin Liu, Ke Pang, Yong-Zhong Tang, Yuan Le

**Affiliations:** Department of Anesthesiology, Third Xiangya Hospital, Central South University, Changsha, China

**Keywords:** pre-operative N-terminal pro-B-type natriuretic peptide, post-operative acute kidney injury, prediction model, non-cardiac surgery, cohort study

In the published article, there was an error in the author list. Authors “Xiang-Bin Liu” and “Ke Pang” were erroneously missing “^†^”, to indicate these authors contributed equally to this work and share the first authorship. The corrected author list appears below.

“Xiang-Bin Liu^†^, Ke Pang^†^, Yong-Zhong Tang^*^ and Yuan Le^*^

Department of Anesthesiology, Third Xiangya Hospital, Central South University, Changsha, China

^*^Correspondence:

Yong-Zhong Tang

tangyongzhong@csu.edu.cn

Yuan Le

leyuanxy@csu.edu.cn

^†^These authors have contributed equally to this work and share first authorship”

The authors apologize for this error and state that this does not change the scientific conclusions of the article in any way. The original article has been updated.

## Publisher's note

All claims expressed in this article are solely those of the authors and do not necessarily represent those of their affiliated organizations, or those of the publisher, the editors and the reviewers. Any product that may be evaluated in this article, or claim that may be made by its manufacturer, is not guaranteed or endorsed by the publisher.

