# Supplementary material for: The Predictive Value of Pre-operative N-Terminal Pro-B-Type Natriuretic Peptide in the Risk of Acute Kidney Injury After Non-cardiac Surgery
Source: Front Med (Lausanne). 2022 Jun 16;9:898513. doi: 10.3389/fmed.2022.898513 (PMC9244627; doi:10.3389/fmed.2022.898513)
Supplement: Supplementary file 1 [file Table_1.DOC]

Supplementary Table S1 Baseline characteristics of the study

| **Variables ^a^** | **Overall** | **No AKI** | **AKI** | **p** |
| --- | --- | --- | --- | --- |
| n | 3949 | 3740 | 209 |  |
| Height ,cm | 159.99 (8.80) | 159.87 (8.85) | 162.15 (7.50) | <0.001 |
| Body weight,kg | 60.48 (12.45) | 60.52 (12.47) | 59.71 (12.07) | 0.356 |
| **Medical history** |  |  |  |  |
| Alcohol(%) | 130 ( 3.3) | 122 ( 3.3) | 8 ( 3.8) | 0.805 |
| tabacco (%) | 205 ( 5.2) | 191 ( 5.1) | 14 ( 6.7) | 0.396 |
| CAD(%) | 787 (19.9) | 734 (19.6) | 53 (25.4) | 0.054 |
| MI (%) | 123 ( 3.1) | 113 ( 3.0) | 10 ( 4.8) | 0.221 |
| Heart failure (%) | 29 ( 0.7) | 27 ( 0.7) | 2 ( 1.0) | 1 |
| hyperlipidemia (%) | 269 ( 6.8) | 259 ( 6.9) | 10 ( 4.8) | 0.292 |
| Diabetes mellitus(%) | 701 (17.8) | 656 (17.5) | 45 (21.5) | 0.169 |
| Renal disease (%) | 317 ( 8.0) | 279 ( 7.5) | 38 (18.2) | <0.001 |
| Pulmonary infection (%) | 634 (16.1) | 564 (15.1) | 70 (33.5) | <0.001 |
| Bronchial diseases(%) | 180 ( 4.6) | 166 ( 4.4) | 14 ( 6.7) | 0.176 |
| Ascites (%) | 76 ( 1.9) | 69 ( 1.8) | 7 ( 3.3) | 0.2 |
| malignancy(%) | 1146 (29.0) | 1090 (29.1) | 56 (26.8) | 0.516 |
| **Meditations 1 week before surgery** |  |  |  |  |
| Anti-hypertension (%) | 635 (16.1) | 581 (15.5) | 54 (25.8) | <0.001 |
| Calcium channel blocker(CCB) (%) | 7 ( 0.2) | 7 ( 0.2) | 0 ( 0.0) | 1 |
| hypoglycemic agent (%) | 24 ( 0.6) | 23 ( 0.6) | 1 ( 0.5) | 1 |
| Insulin (%) | 261 ( 6.6) | 229 ( 6.1) | 32 (15.3) | <0.001 |
| Anti-arrhythmic (%) | 122 ( 3.1) | 109 ( 2.9) | 13 ( 6.2) | 0.013 |
| Hypolipidemic(%) | 101 ( 2.6) | 94 ( 2.5) | 7 ( 3.3) | 0.603 |
| Cardiotonics (%) | 42 ( 1.1) | 34 ( 0.9) | 8 ( 3.8) | <0.001 |
| Anti-depression and anti-anxiety (%) | 127 ( 3.2) | 113 ( 3.0) | 14 ( 6.7) | 0.006 |
| **Pre-operative findings** |  |  |  |  |
| General anesthesia(%) | 3162 (80.1) | 2989 (79.9) | 173 (82.8) | 0.359 |
| ASA 1 (%) | 54 ( 1.4) | 54 ( 1.4) | 0 ( 0.0) |  |
| Diastolic BP，mmHg | 77.91 (9.66) | 77.95 (9.59) | 77.24 (10.90) | 0.305 |
| Systolic BP, mmHg | 130.40 (15.68) | 130.30 (15.61) | 132.17 (16.73) | 0.094 |
| latest Systolic BP before anaesthesia, mmHg | 130.09 (18.34) | 130.03 (18.19) | 131.24 (20.91) | 0.35 |
| WBC,10^9^L^-1^ | 7.90 (6.98) | 7.81 (7.06) | 9.39 (5.31) | 0.001 |
| RBC, 10^12^L^-1^ | 4.07 (0.67) | 4.10 (0.66) | 3.64 (0.79) | <0.001 |
| ALT, U L^-1^ | 26.70 (35.51) | 25.97 (31.69) | 39.71 (75.56) | <0.001 |
| AST, U L^-1^ | 27.40 (29.00) | 26.71 (23.97) | 39.78 (74.00) | <0.001 |
| TP,g L^-1^ | 65.15 (7.49) | 65.40 (7.28) | 60.66 (9.51) | <0.001 |
| GLB, g L^-1^ | 26.60 (4.99) | 26.62 (4.94) | 26.23 (5.86) | 0.276 |
| A/G | 1.53 (0.64) | 1.53 (0.65) | 1.39 (0.36) | 0.001 |
| TBil,μmol L^-1^ | 15.46 (17.99) | 15.04 (15.64) | 22.84 (41.07) | <0.001 |
| DBil,μmol L^-1^ | 5.83 (13.13) | 5.47 (11.11) | 12.28 (31.77) | <0.001 |
| TBA,μmol L^-1^ | 6.38 (14.67) | 6.22 (14.30) | 9.14 (20.00) | 0.005 |
| Urea, mmol L^-1^ | 5.55 (2.85) | 5.44 (2.49) | 7.54 (6.23) | <0.001 |
| eGFR,ml min^-1^ 1.73 m^-2^ (mean (SD)) | 88.99 (22.78) | 89.56 (22.06) | 78.78 (31.53) | <0.001 |
| proteinuria (%) |  |  |  | <0.001 |
| 0 | 3098 (78.5) | 2980 (79.7) | 118 (56.5) |  |
| 1+ | 392 ( 9.9) | 359 ( 9.6) | 33 (15.8) |  |
| 2+ | 229 ( 5.8) | 202 ( 5.4) | 27 (12.9) |  |
| 3+ | 139 ( 3.5) | 121 ( 3.2) | 18 ( 8.6) |  |
| 4+ | 88 ( 2.2) | 75 ( 2.0) | 13 ( 6.2) |  |
| 5+ | 3 ( 0.1) | 3 ( 0.1) | 0 ( 0.0) |  |
| **Surgical characteristics** |  |  |  |  |
| Lowest MAP, mmHg | 61.66 (16.35) | 61.98 (16.26) | 55.93 (17.01) | <0.001 |
| Highest MAP, mmHg | 122.82 (21.58) | 122.51 (21.19) | 128.37 (27.01) | <0.001 |
| Lowest Systolic BP, mmHg | 83.23 (25.47) | 83.67 (25.31) | 75.35 (27.01) | <0.001 |
| Highest Systolic BP mmHg | 173.76 (32.91) | 173.20 (32.59) | 183.87 (36.89) | <0.001 |
| Lowes Diastolic BP, mmHg | 47.39 (14.59) | 47.68 (14.52) | 42.34 (14.93) | <0.001 |
| Highest Diastolic BP, mmHg | 102.18 (21.56) | 101.92 (21.19) | 106.90 (27.02) | 0.001 |
| Infusion volume,ml | 2063.67 (1141.18) | 2058.75 (1139.60) | 2151.62 (1168.49) | 0.252 |
| Blood transfusion,ml | 185.16 (495.27) | 170.62 (479.92) | 445.27 (666.14) | <0.001 |
| Vasoactive meditations (%) | 903 (22.9) | 812 (21.7) | 91 (43.5) | <0.001 |
| norepinephrine (%) | 567 (14.4) | 498 (13.3) | 69 (33.0) | <0.001 |
| Operation scale (%) |  |  |  | 0.008 |
| 1 | 27 ( 0.7) | 26 ( 0.7) | 1 ( 0.5) | |
| 2 | 658 (16.7) | 641 (17.1) | 17 ( 8.1) | |
| 3 | 2819 (71.4) | 2653 (70.9) | 166 (79.4) | |
| 4 | 445 (11.3) | 420 (11.2) | 25 (12.0) | |
| Type of surgery(%) |  |  |  | <0.001 |
| General surgery | 1624 (41.1) | 1537 (41.1) | 87 (41.6) | |
| Gynecology | 790 (20.0) | 779 (20.8) | 11 ( 5.3) | |
| Obstetrics | 111 ( 2.8) | 106 ( 2.8) | 5 ( 2.4) | |
| Orthopedics | 728 (18.4) | 683 (18.3) | 45 (21.5) | |
| Neurosurgery | 308 ( 7.8) | 277 ( 7.4) | 31 (14.8) | |
| Vascular Surgery | 103 ( 2.6) | 93 ( 2.5) | 10 ( 4.8) | |
| Thoracic surgery | 79 ( 2.0) | 73 ( 2.0) | 6 ( 2.9) | |
| Gastroenterology | 44 ( 1.1) | 40 ( 1.1) | 4 ( 1.9) | |
| Interventional procedures | 24 ( 0.6) | 21 ( 0.6) | 3 ( 1.4) | |
| Stomatology | 6 ( 0.2) | 6 ( 0.2) | 0 ( 0.0) | |
| Ophthalmology | 1 ( 0.0) | 1 ( 0.0) | 0 ( 0.0) | |
| ENT | 77 ( 1.9) | 75 ( 2.0) | 2 ( 1.0) | |
| Other | 54 ( 1.4) | 49 ( 1.3) | 5 ( 2.4) | |

Values are mean (SD) or number (%). a Categorical variables were shown as counts (percentages) and continuous variables as means (Standard Deviation).; AKI, acute kidney injury，CHD, coronary heart disease; MI, myocardial infarction; ASA, American Society of Anesthesiologists; WBC,white blood cell count,RBC,red blood cell count;ALT, Alaninetransaminase ;AST, Aspartate Transaminase ; TP, total protein; GLB,globulin; A/G,albumin/globulin; TBil, Total bilirubin; DBil direct bilirubin,; TBA, Total bile acid; eGFR, estimated glomerular fifiltration rate; ENT, Ears, Nose and Throat

Supplimentary Table S2 The relationship between pre-operative pro-BNP measurement and development of AKI in different types of surgery

| Type of surgery | **Overall** | **No AKI** | **AKI** |  | **Median NT-proBNP ng L-1(IQR)** | **OR (95% CI)** |  |
| --- | --- | --- | --- | --- | --- | --- | --- |
| General surgery(%) | 1624 (41.1) | 1537 (41.1) | 87 (41.6) | | 271.81 (127.84 to 642.43) | 1.84（1.56 to 2.19） |  |
| Gynecology(%) | 790 (20.0) | 779 (20.8) | 11 ( 5.3) | | 158.23 ( 73.69 to 347.79 ) | 1.54（0.89 to 2.25 ） |  |
| Orthopedics(%) | 728 (18.4) | 683 (18.3) | 45 (21.5) | | 300.75 (124.47 to 873.79 ) | 1.36（1.16 to 1.59 ） |  |
| Neurosurgery(%) | 308 ( 7.8) | 277 ( 7.4) | 31 (14.8) | | 698.14 (269.00 to 1548.60 ) | 1.32（1.09 to 1.62） |  |
| Other(%) | 499 (12.6) | 464 ( 12.4) | 35 (16.7) | | 309.19 (138.19 to 993.35 ) | 1.42（1.22 to 1.68） |  |

AKI, acute kidney injury；NT-proBNP, N-terminal pro- B-type natriuretic peptide；IQR，Interquartile Range；OR,Odds Ratio;95% CI,95% confidence interval
